# Hyperstable U1snRNA complementary to the K-ras transcripts induces cell death in pancreatic cancer cells

**DOI:** 10.1038/sj.bjc.6600563

**Published:** 2002-10-07

**Authors:** K Kato, Y Hitomi, K Imamura, H Esumi

**Affiliations:** Investigative Treatment Division, National Cancer Center Research Institute East, 6-5-1, Kashiwanoha, Kashiwa, Chiba 277-8577, Japan

**Keywords:** pancreatic cancer, U1 small nuclear RNA, K-ras

## Abstract

One of the critical steps that governs the inhibitory effect of antisense RNA on target gene expression is the association of the antisense RNA with the target RNA molecules. However, until now, no systematic method has been available to select the suitable parts of a gene as antisense targets. In this study, we utilised U1 small nuclear RNA (snRNA) that binds physiologically to the 5′ splice site (5′ss) of pre-mRNA, to develop a novel vector system that permits imposed binding of antisense RNA to its target. The 5′ free end of U1snRNA was replaced with the antisense sequence against the K-ras gene to generate a hyperstable U1snRNA, whose binding stability to 5′ss of the K-ras transcript is ten-fold higher than that of wild-type U1snRNA. The efficacy of such hyperstable U1snRNA was examined by transducing the expression plasmids into human pancreatic cancer cell lines. This revealed that two of the hyperstable U1snRNAs induced cell death after gene transduction, and significantly reduced the number of G418-resistant colonies to less than 10% of the controls. Furthermore, hyperstable U1snRNA suppressed intraperitoneal dissemination of pancreatic cancer cells *in vivo*. Hyperstable U1snRNA might be a novel approach to express effective antisense RNA in target cells.

*British Journal of Cancer* (2002) **87**, 898–904. doi:10.1038/sj.bjc.6600563 www.bjcancer.com

© 2002 Cancer Research UK

## 

Suppression of undesirable gene expression is one important object of gene therapy. It is of particular importance in the development of gene therapy against cancer, in which activated oncogenes play a critical role in neoplastic propagation. A large body of knowledge has accumulated which demonstrates that both antisense oligonucleotides, as well as antisense expression vectors against an activated oncogene, can suppress the transformed phenotypes and tumorigenicity of cancer cell lines (Carter and Lemoine, 1993; Aoki *et al*, 1995, 1997; Gewirtz *et al*, 1998; Kita *et al*, 1999). Since systemic administration of an antisense oligonucleotide has adverse effects due to its non-specific toxicity (Agrawal and Zhao, 1998a,b), it is an urgent task to develop a more efficient antisense vector system and to improve the technique of introducing a foreign gene into target cells.

For the expression of antisense RNA *in vivo*, various vector systems have been developed using RNA polymerase II or III dependent promoters (Strauss, 1997). Although the RNA polymerase II (pol II)-dependent promoter has been used widely, it has disadvantages. For example, the transcribed antisense product is processed as mRNA, polyadenylated, and exported from the nucleus to the cytoplasm where it is eventually degraded. Such a process could be partly responsible for the fact that a short antisense RNA transcribed by pol II was relatively unstable (He and Huang, 1997). The pol III-dependent promoters of small RNA species, such as tRNA and U6snRNA, have been an attractive alternative (Konforti *et al*, 1993; Noonberg *et al*, 1994). However, the use of these pol III-dependent promoters limits the design of antisense RNA, because pol III transcription was found to be impeded by several sequences, which prematurely terminated the transcription (He *et al*, 1997; Das *et al*, 1988).

Despite many efforts, a general algorithm for choosing the best antisense targets has not yet been established. This is partly because there is no method to predict the accessibility of antisense RNA to the target site in the secondary structure of an RNA molecule. Furthermore, mRNA forms a complex with proteins in both nucleus and cytosol (Larson and Sells, 1987; Dreyfuss *et al*, 1993), which makes the selection of an effective antisense sequence largely a matter of trial and error (Birikh *et al*, 1997; Strauss, 1997; Gewirtz *et al*, 1998).

Among the small nuclear RNAs (snRNA), U1snRNA is the most abundant snRNA that is transcribed by pol II. It participates in the recognition of 5′ splice site (5′ss) during pre-mRNA splicing by binding to the 5′ss consensus sequence. This interaction is mediated by the 5′-free end of U1snRNA, which has a 9 base-long complementary sequence to the consensus (Mount *et al*, 1983; Sharp, 1994; Black, 1995). It has been well documented that the introduction of mutations in this 5′-free end can alter the binding specificity of U1snRNA to the 5′ss (Zhuang and Weiner, 1986; Hitomi *et al*, 1998).

These properties of U1snRNA, especially the physiological binding to pre-mRNA, could render it beneficial as a carrier of an antisense sequence against a target transcript, if the sequence at the 5′-free end could be replaced with an antisense sequence. The remaining U1snRNA sequence might stabilise the antisense RNA as a ribonucleoprotein complex, guide it to the pre-mRNA of the target gene, and facilitate association of the antisense RNA to the target site. In addition to conventional antisense effects, if an antisense sequence was long enough to far exceed the binding stability between wild-type U1snRNA and pre-mRNA, it is possible that such hyperstable U1snRNA might disturb the splicing of the target gene transcripts.

In this study, we constructed an antisense expression plasmid based on the U1snRNA gene, and designed hyperstable U1snRNA against the human K-ras transcript. The efficacy of the hyperstable U1snRNA was examined using three pancreatic cancer cell lines, which have been employed previously to demonstrate the inhibitory effects of K-ras antisense oligonucleotides (Kita *et al*, 1999) or expression vectors (Aoki *et al*, 1995, 1997).

## MATERIALS AND METHODS

### Plasmids

**Figure 1:**
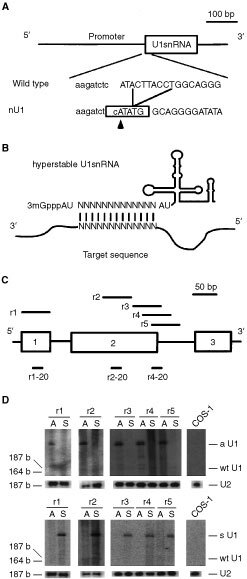
Construction of the hyperstable U1snRNA vector. (**A**) In the human U1snRNA gene (Hitomi *et al*, 1998), six- bases were deleted to generate a NdeI site in the region of the 5′-free end of U1snRNA. The resulting sequence in this region (nU1) is shown with respect to the wild-type sequence. The newly created NdeI site is boxed and the arrowhead indicates the cleavage site. (**B**) Insertion of the antisense sequence in the U1snRNA gene generates hyperstable U1snRNA, which binds to the target site via the inserted sequence in the 5′-free end of U1snRNA. The secondary structure of U1snRNA is shown as a line drawing. (**C**) The target sites against the human K-ras transcript are indicated as r1, r2, r3, r4 and r5, aligned to the corresponding K-ras gene structure. The K-ras exons 1, 2, and 3 are indicated by the numbered boxes. The sites of the 20 base-long oligonucleotides used for northern analysis are also indicated as r1-20 for the detection of r1, r2-20 for r2, r4-20 for r3, r4, and r5. The relative lengths of the target sites, exons, and oligonucleotides are fitted to the scale shown above, whereas the intron sequences are shortened. (**D**) Transient expression of hyperstable U1snRNA in COS-1. The expression plasmid of hyperstable U1snRNA was transfected into COS-1. Three μg of total RNA was separated on a 5% polyacrylamide/7 M urea gel, electroblotted and hybridised with radiolabelled sense (upper panels) or antisense (lower panels) K-ras oligonucleotides corresponding to the target sites, r1 to r5. The position of antisense hyperstable U1snRNA (aU1), sense hyperstable U1snRNA (sU1), and wild-type U1snRNA (wtU1, 164 bases) is indicated. The integrity and load of RNA was examined by hybridizing the same membrane with U2B, an U2snRNA-specific oligonucleotide.

The U1snRNA expression vector has been described elsewhere (Hitomi *et al*, 1998). The *Nde*I site was created within the region of the 5′-free end of U1snRNA, using mutated oligomers GAT CTC ATA TGG CAG GGG AGA TAC and TCC CCT GCC ATA TGA GAT CTT GG, generating pEUK/nU1. Antisense and sense oligomers of five target sequences in the K-ras gene (see Figure 1C

) were annealed and ligated with pEUK/nU1 digested with *Nde*I. The orientation of the insert was confirmed by sequencing, and plasmids were designated, according to the orientation of the insert, as r1A or r1S for antisense or sense against the r1 target site, respectively. The neomycin-resistant gene was subcloned between *Xho*I and *Hind*III sites in pEUK/nU1, where it was under the control of SV40 late promoter.

### Cell culture

Pancreatic carcinoma cell lines, PANC-1, AsPC-1 and BxPC-3 were cultured in DMEM with 10% foetal calf serum (FCS), RPMI-1640 with 20% FCS, and RPMI-1640 with 10% FCS under 5% CO_2_ at 37°C, respectively. COS-1 was cultured in DMEM with 10% FCS. PANC-1 and AsPC-1 have a point mutation in K-ras codon 12, while BxPC-3 has the wild-type K-ras gene (Berrozpe *et al*, 1994)

### Transfection

COS-1 was electroporated with 10 μg of the plasmid at 300 V and 960 μF. Transfection for pancreatic cancer cell lines was performed with transfectum or lipofectamine reagents (GibcoBRL, Rockville, MD, USA) as described by Aoki *et al* (1995). Briefly, the day before transfection, 1×10^5^ cells were plated in a 6 cm dish. DNA and the transfection reagent in serum-free Opti-MEM (GibcoBRL) were overlaid onto the cells. The transfection was halted at 6 h for PANC-1 and AsPC-1, and at 3.5 h for BxPC-3. Transfection efficiency was always monitored by introducing pEGFP-C1 in parallel and examining the green fluorescence microscopically. To establish permanent transfectants, selection with G418 was started 48 h after transfection. After selection for 2 weeks, the number of colonies was counted, and each colony was cloned in the case of r1A and r3A transfectants. For the other transfectants, G418-resistant colonies were pooled and maintained as a mixed population of G418-resistant cells.

### Northern blot analysis

Total RNA was extracted by acid guanidinium thiocyanate-phenol-chloroform extraction (Chomczynski and Sacchi, 1987). Three μg of total RNA was electrophoresed on a denaturing 5% polyacrylamide gel containing 7 M urea, and stained with 5 μg ml^−1^ of ethidium bromide. The fractionated RNA was electroblotted onto a nylon membrane, hybridised with appropriate 20 base-long oligonucleotides to detect an antisense or sense K-ras sequence in hyperstable U1snRNA, and visualised by exposure against an imaging plate (BAS-2000 system, Fuji, Japan). The membrane was also hybridised with an U1snRNA-specific oligomer CCA CCT TCG TGA TCA T, and then U2B, an U2snRNA-specific oligomer AAC AGA TAC TAC ACT TG.

### Immunoblotting

Cells were lysed directly in buffer containing 100 mM Tris-HCl (pH 6.8), 2.5% sodium dodecylsulfate, 20% glycerol, 2 mM phenylmethylsulphonyl fluoride, 2 mM β-mercaptoethanol, and 0.005% bromophenol blue. Total cell lysates (100 μg protein) were heated at 100°C, separated by 12% denaturing polyacrylamide gel, and electroblotted onto polyvinylidene difluoride membrane (Millipore, Bedford, MA, USA). The K-ras protein was detected by a K-ras-specific p21 monoclonal antibody (Oncogene Research Products, Boston, MA, USA) by using enhanced chemiluminescence system (Amersham Pharmacia Biotech, Upsala, Sweden).

### *In vivo* tumorigenicity assay

The tumorigenicity of stable PANC-1 transfectants of the hyperstable U1snRNA plasmid was examined by injecting 3×10^6^ cells subcutaneously into 6 week-old male BALB/c nu/nu mice. Tumour formation was monitored for 3 months. The volume of each tumour was approximated by the formula: tumour volume=r^2^×l/2, where *r* and *l* are two orthogonal measurements of tumour diameter (Gray *et al*, 1993).

### Effects of hyperstable U1snRNA on the peritoneal dissemination model

AsPC-1 of 5×10^5^ cells was injected intraperitoneally into three 6 week-old male BALB/c nu/nu mice in each experimental group (Aoki *et al*, 1995). Three days after tumour cell injection, 100 μg of the hyperstable U1snRNA plasmid was mixed with lipofectamine (GibcoBRL, USA) and injected intraperitoneally three times at 12 h intervals. The mice were sacrificed 28 days after the cell injection and examined for the development of peritoneal dissemination.

## RESULTS

### Construction of the hyperstable U1snRNA plasmids

To develop the novel antisense expression vector based on the U1snRNA gene, a cloning site was created in the U1snRNA gene, which allowed the insertion of any desirable sequence into the 5′-free end of U1snRNA (Figure 1A,B). To determine the maximum length of a sequence that can be stably expressed in conjunction with U1snRNA, double-stranded nucleotides of various lengths ranging from 20 to 100 bases long were inserted into the U1snRNA gene, and the generated plasmids were introduced transiently into COS-1. Northern analysis showed that the hyperstable U1snRNAs acquiring the 60 base-long insert were expressed at comparable levels to those having only 20 to 40 base inserts, whereas the expression levels of the 80 to 100 base inserts seemed to depend on the sequence joined to U1snRNA (data not shown).

Based on these findings, several 60 base-long sequences from the human K-ras gene were selected to examine the efficacy of the hyperstable U1snRNA (Figure 1C). The sequence of r1 was designed to cover the short exon 1 sequence including both 3′ and 5′ss regions. The r3, r4, and r5 sequences spanned the 5′ splice site of the second intron. Their sequences were each shifted 20 bases from one another, while the r2 sequence was in the middle region of exon 2 (see Figure 1C). The binding stability of these 60 base-long RNAs ranges from −85.6 to −115.8 kcal mol^−1^, which is about 10 times higher than that of wild-type U1snRNA (Freier *et al*, 1986; Hitomi *et al*, 1998).

These hyperstable U1snRNAs were transfected into COS-1 transiently and the expression was examined by Northern hybridisation with K-ras-specific oligomers. All types of hyperstable U1snRNA were expressed at similar levels and showed identical lengths (Figure 1D). The relative mobility of these bands, compared to the wild-type U1snRNA and U2snRNA, was consistent with an expected length of about 60 bases longer than the wild-type U1snRNA. These bands could also be detected with an U1snRNA-specific antisense oligomer, which indicated that these bands actually consisted of the K-ras-specific sequence and U1snRNA (data not shown).

### Reduction of G418-resistant colony formation by r1A and r3A hyperstable U1snRNAs

To investigate the effect of hyperstable U1snRNA on cell growth of a pancreatic cancer cell line that expressed the activated K-ras gene, these constructs were transfected into PANC-1. Two days after transfection, we noticed that the cell density was slightly lower in the dishes transfected with r1A and r3A compared to the control dishes transfected with hyperstable U1snRNAs having the sense orientation of the K-ras sequence or wild-type U1snRNA (data not shown). The reduction in the number of attached cells after r1A or r3A transfection could have resulted from an interference with cell attachment, suppression of proliferation, or induction of cell death. The first possibility was discarded in part by examining the degree of trypan blue exclusion in the floating cells after transfection. It showed that all cells were readily stained with the dye. Thus, considering transfection efficiency of these cell lines, which is at most 35%, almost all of the cells transfected with r1A or r3A should slow or cease their proliferation immediately after transfection.

**Figure 2:**
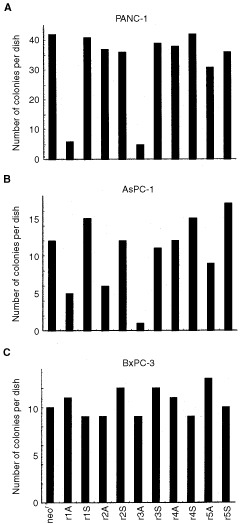
Effect of hyperstable U1snRNA on cell growth and colony formation of pancreatic cancer cell lines. Three pancreatic cancer cell lines, PANC-1 (**A**), AsPC-1 (**B**), and BxPC-3 (**C**) were plated at 1×10^5^ cells in each 6 cm dish and transfected with the hyperstable U1snRNA plasmids having the neomycin-resistant gene (neo^r^). The vehicle vector was also transfected to establish control cells. The number of developed colonies was counted after 2-weeks selection with G418.

To examine this possibility, the neomycin-resistant gene was subcloned into the hyperstable U1snRNA constructs and selected the G418-resistant transfectants of PANC-1, AsPC-1, and BxPC-3. If r1A and r3A were able to retard cell proliferation, permanent transfectants should form smaller colonies than control transfectants. Surprisingly, after 2-weeks selection, the number of G418-resistant colonies was significantly decreased in r1A and r3A transfectants, in both PANC-1 and AsPC-1 (Figure 2A,B

). Furthermore, several colonies transfected with r1A and r3A were significantly smaller than others. Since such differences were not observed with BxPC-3 transfectants (Figure 2C), it is unlikely that co-expression of the hyperstable U1snRNA simply interfered with the expression of the neomycin-resistant gene. These observations were confirmed by repeating the experiments; they suggested that r1A and r3A interfered specifically with the survival of pancreatic carcinoma cells that harbored the activated K-ras gene.

### Analysis of stable transfectants of r1A or r3A hyperstable U1snRNA

The induction of cell death so soon after transfection made it difficult to analyse the underlying mechanism biochemically using the transiently transfected cells. However, since the size of several colonies selected with G418 was apparently smaller in r1A and r3A than in the controls, we speculated that these surviving cells expressed relatively low levels of U1snRNA, which permitted colony formation. Thus, we characterised these clones of PANC-1 cells transfected with r1A and r3A (see Table 1

**Table 1:**
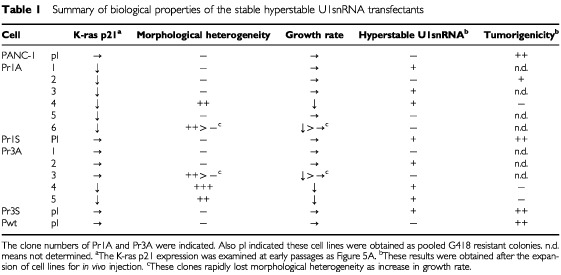
Summary of biological properties of the stable hyperstable U1snRNA transfectants

). Among these clones, two out of six r1A-transfected PANC-1 clones (Pr1A) and three out of five Pr3A clones were quite heterogeneous in morphology, and grew three to four times more slowly than other colonies (Table 1 and Figure 3

**Figure 3:**
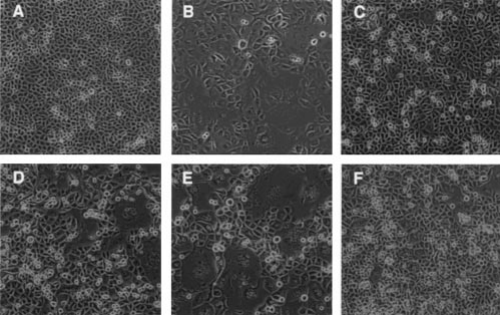
Cell morphology of PANC-1 clones transfected with hyperstable U1snRNA. Parent PANC-1 (**A**) and transfectants, Pr1A-4 (**B**), Pr1S (**C**), Pr3A-4 (**D**), Pr3A-5 (**E**), and Pr3S (**F**) were cultured to confluence and photographed at a magnification of ×200. The r1A and r3A transfected clones are heterogeneous in their cell morphology and contain many multinucleated giant cells.

). These slow growing colonies contained significant numbers of multinucleated giant cells (see Figure 3B,D,E) and also round cells with a dark pyknotic nucleus. Furthermore, cell culture of these clones was always accompanied by a significant number of floating cells that could be stained readily with trypan blue, even after replacing with fresh medium. These features were not observed in PANC-1 colonies transfected with r1S, r3S, wild-type U1snRNA, or the neomycin-resistant gene only. All colonies grown in these control dishes were uniform in size and morphology, which were indistinguishable from those of the parent cell line. During subsequent cell culture for biochemical analysis, Pr1A-6 and Pr3A-3 gradually lost their morphological heterogeneity with their cells becoming more like the parent PANC-1 cell, whereas cell clones Pr3A-4, Pr3A-5, and Pr1A-4 retained their morphological heterogeneity (Table 1). As shown in Figure 4A

**Figure 4:**
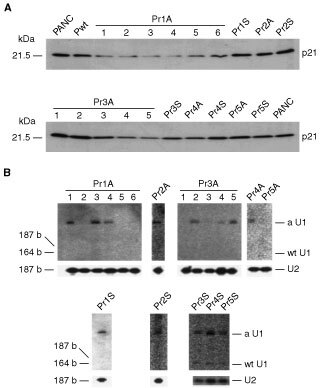
(**A**) Western blot analysis of p21 K-ras proteins. Total cell lysates (100 μg), prepared from parent and stable transfectants at early passage, were subjected to 12% SDS–PAGE, transferred to a membrane, and blotted with K-ras p21-specific monoclonal antibody. (**B**) Expression of hyperstable U1snRNA in PANC-1. The cells were transfected with the hyperstable U1snRNA plasmids and selected with G418. Total RNA (3 μg) was separated on a 5% polyacrylamide/7 M urea gel, blotted on a membrane, and hybridised with radiolabelled sense (upper panels) or antisense (lower panels) K-ras oligonucleotides corresponding to the target sites r1 to r5 (see Figure 1B). The position of antisense hyperstable U1snRNA (aU1), sense hyperstable U1snRNA (sU1), and wild-type U1snRNA (wtU1, 164 bases) is indicated. Hybridisation with U2B verified the integrity and load of RNA.

, immunoblot analysis using the minimum number of cells at early passage indicated that K-ras p21 expression was lower in all Pr1A clones and in two of the Pr3A clones (Pr3A-4 and Pr3A-5) than in the other transfectants. However, such a difference could not be detected after multiple cell passages.

### Suppression of tumorigenicity in the stable r1A or r3A transfectants

**Figure 5:**
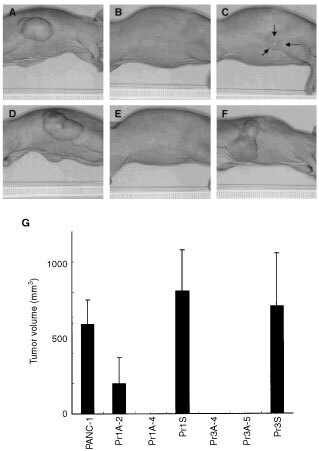
Tumorigenicity of PANC-1 cell lines stably transfected with the expression plasmids of hyperstable U1snRNAs. PANC-1 (**A**), Pr1A-4 (**B**), Pr1A-2 (**C**), Pr1S (**D**), Pr3A-5 (**E**), and Pr3S (**F**). Cells (3×10^6^) were injected subcutaneously into 6 week-old male BALB/c nu/nu mice. The animals were monitored for tumour formation over 3 months and photographed. No tumour developed with Pr1A-4 (**B**) and Pr3A-5 (**E**). Arrows in (**C**) indicate a tumour contour. (**G**) Tumour volume in nude mice was calculated as described (Gray *et al*, 1993) at 3 months following subcutaneous injection.

Expression of hyperstable U1snRNA was examined by Northern analysis. It revealed that the expression of hyperstable U1snRNA was detected in half of these clones, including Pr1A-4, Pr3A-4, and Pr3A-5 (Figure 4B). To investigate the significance of hyperstable U1snRNA expression, we tested the tumorigenicity of these clones. Three of the poor-growing and U1-positive clones (Pr1A-4, Pr3A-4, and Pr3A-5) and one rapidly growing and U1-negative clone (Pr1A-2) were injected in nude mice subcutaneously, in parallel with Pr1S, Pr3S, Pwt, and PANC-1. Two weeks after tumour injection, small nodules were palpable and these gradually enlarged in the control groups. After 3 months, no tumour formation was found in Pr1A-4, Pr3A-4 (Figure 5B,E

), and Pr3A-5 (data not shown), even though the tumours in the control animals injected with parent cells (Pr1S or Pr3S) had enlarged to more than 60 mm^3^ (Figure 5A,D,F,G). In contrast, Pr1A-2 formed tumours that were about one-third the volume of the parent cell tumours (Figure 5C,G). Therefore, the expression of hyperstable U1snRNA appeared to suppress the tumorigenicity of PANC-1.

### Suppression of intraperitoneal dissemination of AsPC-1 by r1A or r3A hyperstable U1snRNA

Finally, the therapeutic effect of r1A and r3A was examined using the abdominal dissemination model of AsPC-1 (Aoki *et al*, 1995). The results are summarised in Table 2

**Table 2:**
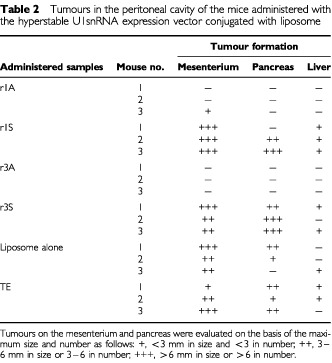
Tumours in the peritoneal cavity of the mice administered with the hyperstable U1snRNA expression vector conjugated with liposome

. Two weeks after cell implantation into the peritoneal cavity, parent AsPC-1 cells formed multiple nodules on the surface of the liver and pancreas, and on the mesentery. However, no tumour nodules were observed in animals that were administered with r1A or r3A, except for a single nodule found on the mesentery of one animal treated with r1A. These findings are in marked contrast to animals injected with r1S or r3S, which showed no essential difference from controls administered with the liposome only or with TE (Table 2). Macroscopically, there was no sign of a general or a local adverse effect of the hyperstable U1snRNA. Therefore, these results indicate that the hyperstable U1snRNA suppressed the peritoneal dissemination of implanted AsPC-1.

## DISCUSSION

A novel plasmid expressing antisense RNA based on the U1snRNA gene has been constructed. The antisense sequence was expressed as hyperstable U1snRNA that exhibited a uniquely high stability of binding with the target RNA. This vector system was able to acquire stably 60 base-long antisense RNA. We tested the efficacy of the vector system by inserting antisense sequences against the human K-ras gene, and found that two of these hyperstable U1snRNAs could induce cell death when transduced into pancreatic cancer cell lines harbouring the activated K-ras oncogene. Furthermore, intraperitoneal administration of these expression plasmids was found to suppress the peritoneal dissemination of implanted pancreatic cancer cells. Although a loss of antisense-transfected cells during clonal expansion has been described, for example, in oesophageal cancer cells with antisense to cyclin D1 (Zhou *et al*, 1995), no such phenomenon has ever been reported using antisense oligomers or antisense expression vectors against the K-ras gene (Mukhopadhyay *et al*, 1991; Zhang *et al*, 1993; Aoki *et al*, 1995, 1997; Alemany *et al*, 1996; Kita *et al*, 1999). The induction of cell death was noticed primarily as a reduction in the number of viable cells following transient transfection of r1A or r3A. This observation was supported by careful cell counting and flow cytometer analysis (data not shown). Further evidence was provided by analysis using the Pr1A and Pr3A clones, obtained as stable r1A and r3A transfectants. Although any comparison between isolated cell clones and pooled cells needs careful interpretation, the colonies of these stable transfectants displayed obvious characteristics, such as a slow growth rate and morphological heterogeneity, which were not observed in the colonies of any control transfectants. The presence of numerous multinucleated giant cells indicated strongly that the hyperstable U1snRNA exerted its effect on the cell cycle, possibly in the mitotic phase rather than S phase, because of the multiple nuclei suggesting that these cells had passed through several rounds of DNA replication. These transfectants also produced a significant number of floating cells, which suggested that a fraction of the transfectants continuously died. Cumulatively, these results provide strong evidence that the hyperstable U1snRNA from r1A and r3A were able to arrest cell proliferation and induce cell death in pancreatic cancer cell lines harbouring the activated K-ras gene.

The induction of cell death so soon after gene transduction hampered biochemical analysis of the underlying mechanism. However, the analysis of stable transfectants provided several clues that suggested that r1A and r3A interfered with the production of K-ras p21. First of all, in the Pr1A and Pr3A clones, there was a correlation between the expression of hyperstable U1snRNA, the reduction of K-ras p21, the morphological heterogeneity, and the loss of tumorigenicity. Furthermore, reversion of cell morphology during cell culture was associated with an elevation in K-ras p21 levels and a decline in the expression of the hyperstable U1snRNA. In addition, the insensitivity of BxPC-3 to these hyperstable U1snRNAs was also consistent with the antisense effect against K-ras, since BxPC-3 was not affected by any antisense strategies in previous studies (Aoki *et al*, 1995, 1997; Kita *et al*, 1999).

The reason for the reduction in K-ras production interfering with cell survival is unknown. However, studies using knockout mice demonstrated that the K-ras gene is essential for certain cell lineages during embryogenesis (Johnson *et al*, 1997; Koera *et al*, 1997). If the survival of pancreatic cancer cells depends to any extent on the expression of an activated K-ras gene, then a reduction in gene expression to less than a certain critical level might be lethal to these cells.

Using hyperstable U1snRNA, one concern is non-specific toxicity arising from the use of a general splicing factor as a carrier of an antisense sequence. Theoretically, hyperstable U1snRNA does have a higher binding stability, but only for its target 5′ss, and not for non-homologous 5′ss regions. Although there is an essential difference with respect to hyperstable U1snRNA, mutated U1snRNAs in the 5′-free end sequence could be expressed stably in culture cells (Cohen *et al*, 1993) and in transgenic flies (Lo *et al*, 1994), suggesting that the cells can tolerate the expression of mutated U1snRNA to a certain extent.

In this study, non-specific toxicity of hyperstable U1snRNA was not observed, since all hyperstable U1snRNAs, except r1A and r3A, did not exert any inhibitory effect on pancreatic cancer cell lines. Even r1A and r3A did not affect cell growth of BxPC-3 and COS-1. Furthermore, *in vivo* injection of a DNA/liposome complex did not produce any recognisable tissue damage in the host animals, while it prevented the peritoneal dissemination of inoculated pancreatic cancer cells. Aoki *et al* (1995) studied the systemic distribution of DNA conjugated with liposome and reported that it could be found in all organs except the brain. Therefore, we considered that these hyperstable U1snRNAs are no more toxic than conventional antisense RNA against the K-ras gene, and might be useful for therapeutic purposes.

We were also surprised by the finding that a shift of merely 20 bases in the target sequences, which still overlapped 40 bases with each other, could abolish the effect of the hyperstable U1snRNA, although it has been known that a slight shift of the target site can affect the inhibitory potency of antisense oligonucleotides. This raises the possibility that the binding of these hyperstable U1snRNAs to the target site may be strictly regulated, in such a way that the 20 base-shift rendered them unable to bind with the target RNA. Alternatively, even though hyperstable U1snRNAs could bind to their target sequences, the outcome of whether they disturb or permit normal processing of the target pre-mRNA may depend on their relative binding sites to the 5′ss. Indeed, the target sites of r1A and r3A were in the 5′ exon side of 5′ss regions. Further study is necessary to determine whether the effect of hyperstable U1snRNA actually depends on its position relative to the 5′ss, and involves an alteration in pre-mRNA processing, as we anticipated before.

Our results demonstrate that the hyperstable U1snRNA is much more effective than conventional antisense RNA against K-ras, despite the fact that we used relatively short antisense sequences as an *in vivo* antisense expression vector. By directing the antisense RNA to the 5′ss region of pre-mRNA in the form of hyperstable U1snRNA, this vector system gives us the potential to select the target sequence systematically in the vicinity of a 5′ss in any gene.
